# AFAP1L1, a novel associating partner with vinculin, modulates cellular morphology and motility, and promotes the progression of colorectal cancers

**DOI:** 10.1002/cam4.237

**Published:** 2014-04-10

**Authors:** Ryo Takahashi, Satoshi Nagayama, Moritoshi Furu, Yoichiro Kajita, YongHui Jin, Tomohisa Kato, Seiya Imoto, Yoshiharu Sakai, Junya Toguchida

**Affiliations:** 1 Department of Tissue Regeneration, Institute for Frontier Medical Sciences, Kyoto UniversityKyoto, Japan; 2 Department of Surgery, Graduate School of Medicine, Kyoto UniversityKyoto, Japan; 3 Gastroenterology Center, The Cancer Institute Hospital of Japanese Foundation of Cancer ResearchTokyo, Japan; 4 Department of Orthopaedic Surgery, Graduate School of Medicine, Kyoto UniversityKyoto, Japan; 5 Department of Urology, Graduate School of Medicine, Kyoto UniversityKyoto, Japan; 6 Center for iPS Cell Research and Application, Kyoto UniversityKyoto, Japan; 7 Laboratory for DNA Information Analysis, Human Genome Center, Institute of Medical Science, The University of TokyoKyoto, Japan

**Keywords:** AFAP1L1, cell motility, colorectal cancer, invadopodia, vinculin

## Abstract

We have previously identified *actin filament-associated protein 1-like 1* (*AFAP1L1*) as a metastasis-predicting marker for spindle cell sarcomas by gene expression profiling, and demonstrated that AFAP1L1 is involved in the cell invasion process by in vitro analyses. However, its precise molecular function has not been fully elucidated, and it remains unknown whether AFAP1L1 could be a prognostic marker and/or therapeutic target of other malignancies. In this study, we found a marked elevation of *AFAP1L1* gene expression in colorectal cancer (CRC) tissues as compared to the adjacent normal mucosa. Multivariate analysis revealed that AFAP1L1 was an independent and significant factor for the recurrence of rectal cancers. Moreover, the addition of the AFAP1L1 expression level to the lymph node metastasis status provided more predictive information regarding postoperative recurrence in rectal cancers. AFAP1L1-transduced CRC cells exhibited a rounded shape, increased cell motility on planar substrates, and resistance to anoikis in vitro. AFAP1L1 localized to the ringed structure of the invadopodia, together with vinculin, and AFAP1L1 was identified as a novel associating partner of vinculin by immunoprecipitation assay. AFAP1L1-transduced cells showed accelerated tumor growth in vivo, presumably reflecting the anoikis resistance of these AFAP1L1-expressing cells. Furthermore, the local administration of a siRNA against AFAP1L1 significantly suppressed the in vivo tumor growth of xenografts, suggesting that AFAP1L1 might be a candidate therapeutic target for CRCs. These results suggest that AFAP1L1 plays a role in the progression of CRCs by modulating cell shape and motility and by inhibiting anoikis, presumably through interactions with vinculin-including protein complexes.

## Introduction

One of the most crucial steps in cancer progression is the acquisition of the ability for tumor cells to invade across the basement membrane and into the surrounding tissues, which leads to metastatic spread and eventual cancer-related death. During this process, tumor cells usually reorganize their preexisting actin cytoskeleton and change their cellular shapes 1. Some motile cells adjust their shapes constantly to the surrounding microenvironment to optimize a migratory mode. There are at least two different modes: mesenchymal and amoeboid migration. Mesenchymal migration is a fibroblast-like movement, depending strongly on the surrounding substrate and sufficient cytoskeletal contractility. In contrast, amoeboid migration refers to the movement of rounded or ellipsoid cells that lack mature focal adhesions and stress fibers 2,3. These cellular morphological changes are often associated with the formation of proteolytic protrusions. Unique protrusions on the ventral membrane with adhesive and degradative properties were termed invadopodia in cancer cell lines 4. The proteolytic activity of these invadopodia is induced mainly by the recruitment of various matrix metalloproteases (MMPs), especially membrane type 1 matrix metalloprotease (MT1-MMP), MMP-2, and/or MMP-9 5–8. These specialized processes for migration and/or invasion are regulated by a number of molecules, including integrins, the Rho-family of small GTPases (Rho-GTPases), focal adhesion kinase (FAK), protein kinase C, and phosphatidylinositols, as well as Src and MMPs 2,3,9.

Actin filament-associated protein 1-like 1 (AFAP1L1) is a member of the AFAP family of proteins which includes the well-investigated actin-bundling adaptor protein AFAP1. Our previous study identified the *AFAP1L1* gene as a prognostic marker for spindle cell sarcomas utilizing a genome-wide cDNA microarray. The downregulation of AFAP1L1 in osteosarcoma cells markedly decreased their invasion capability in matrix gels, and the ectopic overexpression of AFAP1L1 in immortalized human mesenchymal stem cells resulted in a significant enhancement of invasiveness. In addition, gelatin zymography demonstrated increased MMP-9 secretion in AFAP1L1-overexpressed cells 10,11. Furthermore, Snyder and coworkers reported the capability of AFAP1L1 to interact with cortactin in invadopodia from breast cancer cell lines 12. These findings suggest that AFAP1L1 plays a role in cell invasion during tumor progression.

However, there is a possibility that AFAP1L1 can exert another tumor-promoting effect. In addition, it is an intriguing issue whether the *AFAP1L1* gene could be a prognostic marker for other malignancies besides sarcomas. As it is known that AFAP1 interacts with both Src and F-actin via its SH3 binding motif and actin-binding domain 13,14, we hypothesized that AFAP1L1 might also interact with other cytoskeleton-related molecules besides cortactin through multiple protein-binding motifs.

In this study, we analyzed *AFAP1L1* gene expression in tissue samples from colorectal cancer (CRC) patients, and assessed its correlation with other clinicopathologic findings. Taking the in vivo analyses into account, we concluded that the *AFAP1L1* gene could be a promising candidate as a biomarker and/or therapeutic target for CRCs. Furthermore, AFAP1L1 was involved in regulating the shape and motility of CRC cells, and was identified as a novel component of vinculin-including complexes by in vitro analyses. We propose an intriguing framework wherein AFAP1L1 plays a part in actin filament remodeling for cellular dynamics, including morphology and motility, partially through its interaction with vinculin.

## Methods

### Cell lines, antibodies, and reagents

All cell lines were obtained from the American Type Culture Collection (Manassas, VA). The anti-AFAP1L1 polyclonal antibody (Ab) was produced in our laboratory as previously described 11. Other Abs and Taqman probes are listed in Table S1.

### Tissue samples

Tumor samples were obtained from 164 CRC patients who had undergone curative resections at the Department of Surgery, Kyoto University Hospital during 1999–2001 for the immunohistochemical analyses using formalin-fixed paraffin-embedded sections, and from 33 and 28 CRC specimens collected during 2006–2007 and 2012 for the reverse transcription (RT)-PCR analyses using frozen samples, respectively. The sixth edition of the tumor-node-metastasis (TNM) classification was used for CRC staging, and the rectal cancers included those located in the recto-sigmoid junction and the upper and lower rectum. All samples were approved for analysis by the Kyoto University Ethics Committee.

### RT-PCR

RNA extraction, reverse transcription, and PCR amplification were performed as previously described 11,15. The expression levels of the target genes were normalized against those of *β*-actin.

### Immunohistochemical analyses

All immunohistochemical experiments were performed as previously described 15. The intensity of the immunostaining was evaluated at the invasion front as 0 (negative), 1 (weak) or 2 (strong) independently by two investigators (R. T. and S. N.) who were blinded to each sample.

### Expression vectors

The human *AFAP1L1* gene was subcloned into a pLenti6/V5-DEST vector (Invitrogen, Carlsbad, CA). As a control, a β-galactosidase gene-expressing vector (pLenti6/V5-GW/LacZ, Invitrogen) was used. Stable cells were selected with blasticidin and several clones were isolated by limiting dilution. For the transient expression, the human *AFAP1L1, AFAP1,* and *vinculin* genes were subcloned into the pcDNA3.1+ plasmid tagged at the N-terminus with 3×Flag or 3×HA (Invitrogen). The transfection was carried out using Lipofectamine 2000 (Invitrogen).

### RNA interference

The siRNA experiments were performed as previously described 16. For the stable knockdown, the shRNA against the *AFAP1L1* gene was cloned into the pLenti6/V5-DEST expression vector (Invitrogen). The cells were then selected with blasticidin and were used without single-cell cloning.

### Time-lapse imaging

5 × 10^4^ cells were seeded onto fibronectin (FN)-coated (10 *μ*g/mL) 24-well plates, and after a 9 h incubation, images were captured every 4 min for 6 h with a CCM-MULTI-KS system (ASTEC, Fukuoka, Japan), and were processed using ImageJ software (NIH, Bethesda, MD) with the Manual Tracking plug-in (http://rsbweb.nih.gov/ij/plugins/track/track.html). All of the cells in random view fields were tracked except for mitotic cells.

### Animal experiments

RKO (2 × 10^6^), LoVo (1 × 10^6^) or SW480 (3 × 10^5^) CRC cells in phosphate buffered saline (PBS) were inoculated subcutaneously (s.c.) into the flanks of 8-week-old male KSN/Slc athymic nude mice (SLC, Shizuoka, Japan). For in vivo siRNA treatment, 1 × 10^6^ cells were inoculated s.c., and at 2 weeks after the inoculation when the tumors had reached a volume of approximately 100 mm^3^, the mice were randomly divided into two treatment groups (AFAP1L1-targeted siRNA and nontargeting siRNA). A stealth RNAi siRNA Negative Control (Invitrogen) was used as the control. The siRNAs were complexed with atelocollagen (Koken, Tokyo, Japan) to a final concentration of 5 *μ*mol/L, and injected into the tumor four times every 5 days. At 3 days after the initial siRNA administration, some tumors were excised to confirm the silencing effect on AFAP1L1 expression levels. At 34 days after the inoculation, all of the tumors were excised and weighed. All experiments with animals were approved by the Animal Research Committee of Kyoto University and were conducted according to the Animal Experiments Guidelines.

### Immunofluorescence microscopy

Immunocytochemistry was performed as previously described 11. The images were obtained with a LSM710 confocal microscopic system (Carl Zeiss, Vienna, Austria) using a 63× oil immersion lens.

### Extracellular matrix-degradation assay

The assays were performed according to published protocols 17. 0.2% gelatin was labeled with Alexa Fluor 488 dye (gelatin-AF488). The cells were then seeded onto gelatin-AF488-coated coverslips in 12-well plates at 1 × 10^5^ cells/well, and were fixed and immunostained 24 h later.

### Western blotting and immunoprecipitation

The analyses were performed as previously described 15,18.

### Anoikis assay

The anoikis assays were performed according to published protocols 19. A total of 5 × 10^5^ cells were suspended in complete media containing 0.5% methylcellulose, and were seeded onto poly-HEMA-coated plates. TdT-mediated dUTP nick end labeling (TUNEL) staining was performed using an in situ Apoptosis Detection Kit (TaKaRa Bio, Shiga, Japan).

### Statistical analyses

Two-sided Student's *t*-tests and chi-square tests were performed between two independent groups. Kaplan–Meier curves were derived to assess the overall and disease-free survivals (DFS), and significant differences in survival times among the patient subgroups were analyzed using log-rank tests. Univariate and multivariate analyses based on logistic regression were used to identify the significant factors relevant to recurrences. Leave-one-out cross-validation was employed to evaluate the predictive power of the AFAP1L1 expression levels for recurrences. Pearson's correlation coefficient was calculated to evaluate the linear dependence between two variables. A *P* value less than 0.05 was considered statistically significant.

## Results

### *AFAP1L1* gene expression was upregulated in CRC tissues

**Figure 1:**
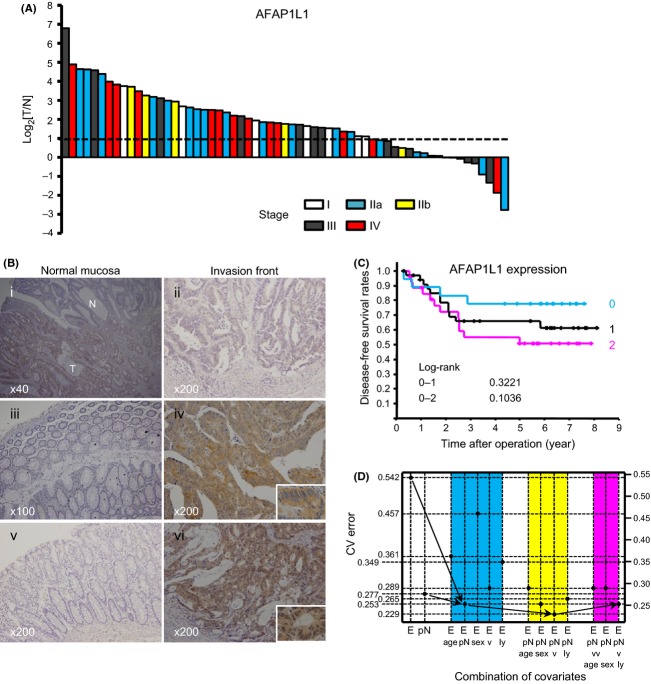
AFAP1L1 expression is upregulated in colorectal cancer tissues. (A) Quantitative analyses of *AFAP1L1* gene expression in CRC tissues and the normal mucosa. The *AFAP1L1* gene expression levels in 61 pairs of CRC tissue specimens and their adjacent normal mucosa were analyzed by qRT-PCR. The AFAP1L1 expression levels were normalized against those of *β*-actin. The ratios of the normalized expressions in the tumors relative to that of the matched normal mucosa in each patient were presented in base two logarithm (log_2_[T/N]). The dashed line indicates log_2_[T/N] = 1. (B) Immunohistochemical analyses for AFAP1L1 expression in CRC tissues and the normal mucosa. Representative images of the normal colonic mucosa (i, iii, v) and at the invasion front of the matched CRCs (ii, iv, vi) from three patients are shown. N and T in (i) indicate the normal mucosa and tumor cells, respectively. Immunoreactivity for AFAP1L1 was defined as negative (i, iii, v), weak (ii) or strong (iv, vi). The original magnifications are indicated in the figures; inset 400×. (C) Kaplan–Meier curves of the disease-free survival (DFS) rates after curative resections for 80 rectal cancers according to their AFAP1L1 expression levels. Differences between the groups were analyzed using the log-rank test, and the *P* values are shown in the figure. (D) Significance of AFAP1L1 as a predictive factor for recurrence. Changes in the cross-validation errors regarding the prediction of recurrence in rectal cancers are shown according to a combination of covariates, including AFAP1L1 expression levels (E), pN status (pN), age, gender, vascular invasion degree (v), and lymphatic invasion degree (ly).

We first analyzed the expression levels of the *AFAP1L1* gene by quantitative RT-PCR (qRT-PCR) using frozen tissues from 33 CRC specimens (two cases at Stage I, 11 at Stage IIa, four at Stage IIb, nine at Stage III, and seven at Stage IV, respectively) along with paired adjacent normal colonic mucosa samples. In 19 of 33 cases, the *AFAP1L1* gene expression levels were upregulated in the tumor tissues more than twofold higher than in the adjacent normal mucosa (log ratio >1.0, Fig. S1A). To confirm this result, a second set of 28 CRC samples were analyzed (five cases at Stage I, nine at Stage IIa, two at Stage IIb, six at Stage III, and six at Stage IV, respectively). Overall, the *AFAP1L1* gene expression levels were upregulated in the tumor tissues in 69% of the cases (42/61, Fig.1A, Table S2). These results were found to be consistent regardless of the different internal controls (*β-actin* or *β-2-microglobulin* (*B2M*) gene expression) (Fig. S1B). In addition, the expression levels of the other members of the AFAP1 family, AFAP1 and AFAP1L2, were examined using the first sample set. The *AFAP1* gene expression levels were increased in the tumor tissues in 36% of the specimens (12/33, Fig. S1C). Although the expression patterns of the *AFAP1* gene were weakly correlated with those of the *AFAP1L1* gene (Pearson's correlation coefficient *r* = 0.408, Fig. S1D), the ratios of the expression levels of the *AFAP1* gene in the tumors versus normal matched samples (T/N) were lower as compared to that of the *AFAP1L1* gene (Fig. S1A and C). On the other hand, the *AFAP1L2* gene expression patterns were random (Fig. S1E), and there were no significant correlations between *AFAP1L1* and *AFAP1L2* gene expressions (*r* = 0.122, Fig. S1F). Taken together, among the AFAP1 family, AFAP1L1 showed the most cancer-specific upregulated expression pattern in CRC tissues.

### The expression of AFAP1L1 was specific to CRC cells

We then proceeded to an immunohistochemical analysis using 164 CRC specimens to evaluate the intratumoral distribution of the AFAP1L1 protein. The patient profiles are summarized in Table S3. Although there was almost no immunoreactivity observed for AFAP1L1 in the normal mucosa, the CRC tissues showed a homogenous immunostaining pattern in the cytoplasm (Fig.1B). There were no marked differences in the immunostaining intensity among the cancer cells. In some slides, there was a tendency for the expression levels to slightly increase in the invasion front as compared to the superficial region. No stromal cells including fibroblasts, macrophages, or endothelial cells were immunostained.

### The expression of AFAP1L1 was associated with the recurrence of rectal cancers

To analyze the significance of AFAP1L1 expression in the CRCs, the expression intensity was compared with respect to various clinicopathologic factors. There were no significant differences detected among any of the factors, except for the significantly increased expression levels of AFAP1L1 in colon cancers (*P* = 0.013, Table S4). Among the 134 curative resections, there was no significant impact on the overall survival or DFS rates according to the expression levels (data not shown). In 80 curative resections for rectal cancers, there was a tendency that the DFS rates were better in those cases immunonegative for AFAP1L1 expression as compared to the strongly immunopositive cases (Fig.1C). Multivariate logistic regression analysis for recurrence in the 80 rectal cancers showed that AFAP1L1 expression was an independent and significant factor, along with the pathological assessment of the regional lymph nodes (pN) status (Table S5). Although the pN status was the most significant factor for recurrence in rectal cancers, a cross-validation analysis showed that a combination of AFAP1L1 expression levels, pN status, and vascular invasion degree provided more predictive information regarding recurrences after curative resections for rectal cancers (Fig.1D).

### AFAP1L1 had no effect on growth in vitro, but correlated positively with tumor growth in vivo

**Figure 2:**
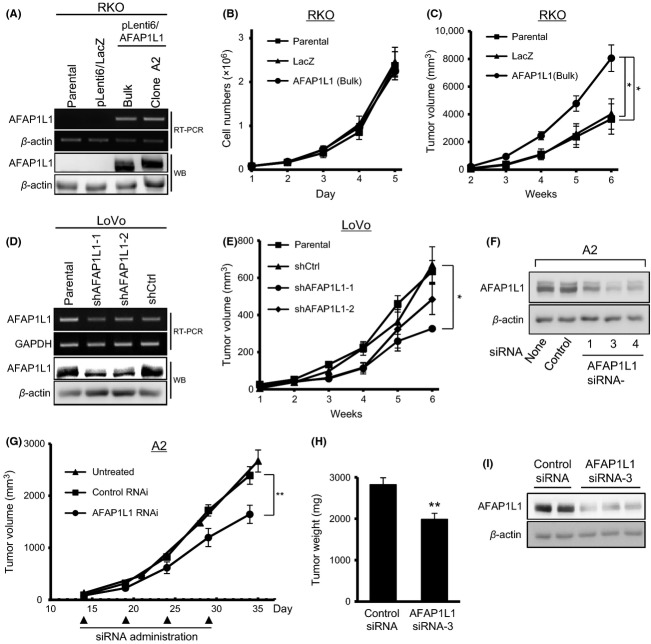
AFAP1L1 expression levels are positively correlated with cell growth in vivo. (A) Established RKO cells stably expressing the exogenous *AFAP1L1* gene and the selected clone A2 cells were analyzed by RT-PCR and immunoblotting. LacZ-transduced cells were used as the control. (B) Growth curves of stable RKO-derived cells in vitro. The indicated cell lines were cultured on 60 mm dishes and were counted with a hemocytometer every 24 h. (C) Growth curves of stable, RKO-derived cells in vivo. The indicated cell lines were inoculated into the flanks of nude mice (*N* = 5 ∼ 8 per group), and were measured by calipers every 7 days. (D) The silencing effects on AFAP1L1 expression levels in stable LoVo cells expressing the indicated shRNA were determined by RT-PCR and immunoblotting. (E) Growth curves of LoVo cells expressing AFAP1L1 or nontargeting shRNA in vivo. Each cell line was inoculated into the flanks of nude mice (*N* = 4 ∼ 6 per group). (F) Knocking-down effects of three different siRNAs against AFAP1L1 and one control siRNA in A2 cells. (G–I) Treatment of A2 xenografts with a siRNA against the *AFAP1L1* gene. On day 14, when the subcutaneously inoculated A2 cells formed a tumor of a specific size, the local administration of control siRNA (*N* = 7) or AFAP1L1 siRNA (*N* = 9) into the xenografts was commenced, and repeated thereafter three times every 5 days. The tumor volume of the siRNA treatment groups and the untreated group (*N* = 5) was monitored during the treatment (G), and the weight of the excised xenografts was measured on day 34 after the inoculation (H). The knocking-down effect of the siRNA was confirmed by immunoblotting using two and three different xenografts from the control group and the experimental group, respectively, at 3 days after the first siRNA administration (I). Each lane represents an independent tumor treated with the indicated siRNA. The data are presented as means ± SE. *P < 0.05; **P < 0.01 by two-sided Student's *t*-test.

We next designed in vitro and in vivo analyses to elucidate the function of AFAP1L1 in CRC cells. Among the 13 CRC cell lines tested, RKO cells were employed as an AFAP1L1-negative cell line, and LoVo cells were selected as the cell line with the highest level of endogenous *AFAP1L1* gene expression (Fig. S2A). We then generated stable AFAP1L1-transduced RKO cells (Fig.2A), in which the expression level of AFAP1L1 was higher than that of LoVo cells. The bulk population (RKO/AFAP1L1) and the established stable clone A2 (A2) were used for further experiments. The in vitro growth of RKO/AFAP1L1 cells showed no significant differences as compared to parental RKO or *β*-galactosidase-transduced control cells (RKO/LacZ) (Fig.2B). However, when inoculated s.c. into nude mice, the xenografts derived from the RKO/AFAP1L1 cells showed significantly more rapid growth (Fig.2C). This result is consistent with our previous finding in the osteosarcoma cell line SaOS2 11. For further confirmation, the same experiment was replicated using two selected stable clones of AFAP1L1-transduced SW480 cells (SW480/AFAP1L1-1 and -2) and LacZ-transduced SW480 cells (SW480/LacZ) (Fig. S2B). Although no differences were observed in terms of in vitro cell growth (data not shown), the growth rates of the in vivo xenografts were increased in proportion to their AFAP1L1 expression levels (Fig. S2C).

Conversely, we also established stable LoVo-derived cells whose endogenous AFAP1L1 protein was knocked down by one of the two targeting sequences (shAFAP1L1-1 and -2) and confirmed that silencing AFAP1L1 does not affect in vitro cell growth (Figs.2D, S2D, and S3A). After inoculating these shRNA-treated LoVo cells, the in vivo tumor growth was significantly attenuated in both LoVo/shAFAP1L1-1 and -2-derived xenografts at 4 weeks after the inoculation, and this tumor growth attenuation persisted in the shAFAP1L1-1-derived tumors until 6 weeks after the inoculation (Fig.2E).

### In vivo growth of RKO/AFAP1L1 was suppressed by the local administration of siRNA against AFAP1L1

We next examined the therapeutic effect of siRNA treatment against AFAP1L1 on the growth of A2-cell-derived xenografts. AFAP1L1 siRNA-3 was used because it had the strongest silencing effect (Figs.2F and S3B). A mixture of siRNA oligonucleotide and atelocollagen was injected locally into palpable xenografts implanted into nude mice. In the AFAP1L1 siRNA-3 treatment group, the tumor growth was significantly inhibited (Fig.2G and H). This downregulation of AFAP1L1 expression following siRNA treatment was confirmed by western blotting using extirpated tumor tissues (Figs.2I and S3C). This result further reinforces the contribution of AFAP1L1 to the in vivo growth of CRC cell-derived xenografts.

### AFAP1L1 regulates cellular morphology and motility on a 2D substrate

**Figure 3:**
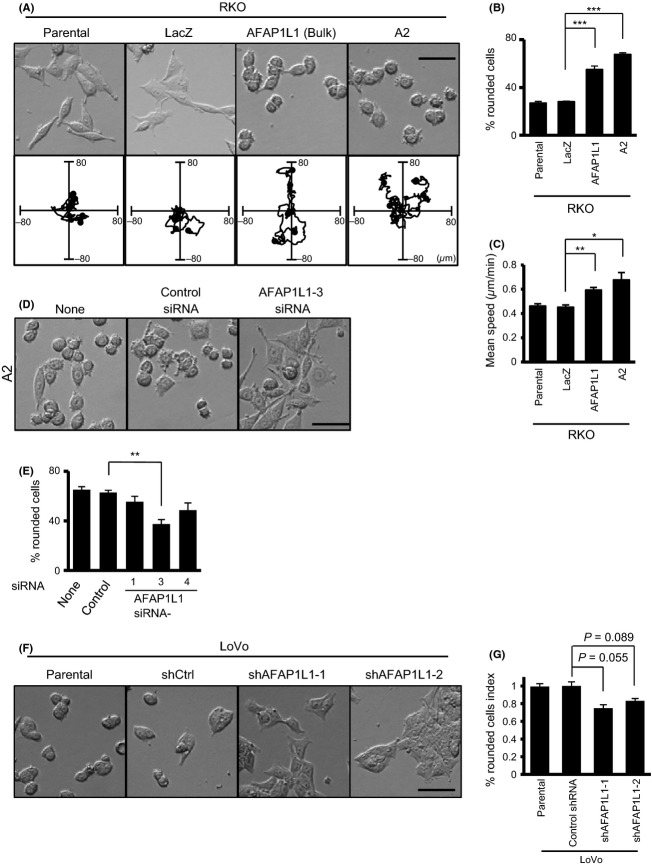
AFAP1L1 expression levels are correlated with cellular morphology and motility. (A) Phase contrast images of stable RKO-derived cells cultured on FN-coated glass slides (upper panel) and representative trajectories of the cellular movements (lower panel) (*N* = 5 for each cell line). (B, C) The proportion of rounded cells (B) and the mean speed of cellular movement (C) of the indicated RKO-derived cells were measured. (D, E) Phase contrast images (D) and the proportion of rounded A2 cells (E) treated without (none) or with the indicated siRNAs. (F, G) Phase contrast images (F) and the proportion of rounded stable LoVo cells (G) expressing the indicated shRNA. The *P* value was calculated by a two-sided Student's *t*-test. The migration speeds were calculated by measuring temporal changes in the positions of the cell nuclei. Approximately 200 cells (177–219) per group were tracked in three independent experiments and the results are presented as means ± SE. *P < 0.05; **P < 0.01; ***P < 0.001 by two-sided Student's *t*-test.

We observed a notable morphological change in RKO cells due to the overexpression of AFAP1L1. The parental RKO and RKO/LacZ cells exhibited either a predominantly flattened or elongated shape. In contrast, the cellular morphology of the RKO/AFAP1L1 and A2 cells was drastically altered to a rounded shape (Fig.3A, upper panels). Furthermore, using time-lapse microscopy, the AFAP1L1-transduced rounded cells were found to move more rapidly on FN-coated dishes (Fig.3A lower panels; movies S1–S4). When quantifying the movement speed, there was a clear correlation between the proportion of rounded cells (Fig.3B) and the mean speed of cellular movement (Fig.3C). Although FN can affect the cell shape and motility, presumably by enhancing cell adhesion to the dishes, the same experiments without FN-coating showed similar results (Fig. S4A and B). We observed no significant differences in the persistence of this cellular movement (Fig. S4C). To confirm the effect of AFAP1L1 on the cell shape, A2 cells were treated with a siRNA against AFAP1L1 (Fig.2F), which then showed a reversion to a flattened morphology (Fig.3D). The proportion of rounded cells decreased in accordance with the silencing efficiency on the AFAP1L1 levels (Fig.2F and E), thus indicating that AFAP1L1 was involved in the regulation of the morphology of CRC cells.

We next investigated the role of endogenous AFAP1L1 in cell morphology control and migration in shRNA-treated LoVo cells. Some of the LoVo/shAFAP1L1-1 and -2 cells showed a flattened morphology (Fig.3F), and there was a substantial but not statistically significant decrease in the proportion of rounded cells after knocking down the AFAP1L1 expression (Fig.3G). However, no significant changes in motility were detected among these cell lines (data not shown). This may be partly due to an insufficient knockdown effect against AFAP1L1 in the LoVo cells, in which a significant amount of AFAP1L1 still functioned.

The tyrosine kinase Src is a well-documented protein that causes cell rounding and enhances motility when activated 20. Moreover, AFAP1 is known to interact with Src, and they can activate each other 21. Thus, we analyzed the levels of activated Src in these cells. The tyrosine 416 phosphorylation levels were almost equal among the stable lines (Fig. S5A). Interestingly, a significant activation of Src was evident in transient transfectants expressing AFAP1, but not AFAP1L1 (Fig. S5B). Moreover, the inhibition of Src activity by 4-amino-5-(4-chlorophenyl)-7-(dimethylethyl) pyrazolo[3,4-*d*]pyrimidine (PP2) treatment resulted in cell rounding (Fig. S5C–E). Taken together, we speculate that the effect on cell shape and motility induced by AFAP1L1 overexpression was not due to a modulation of Src activation.

### AFAP1L1 was clustered in the ringed structure of the invadopodia

**Figure 4:**
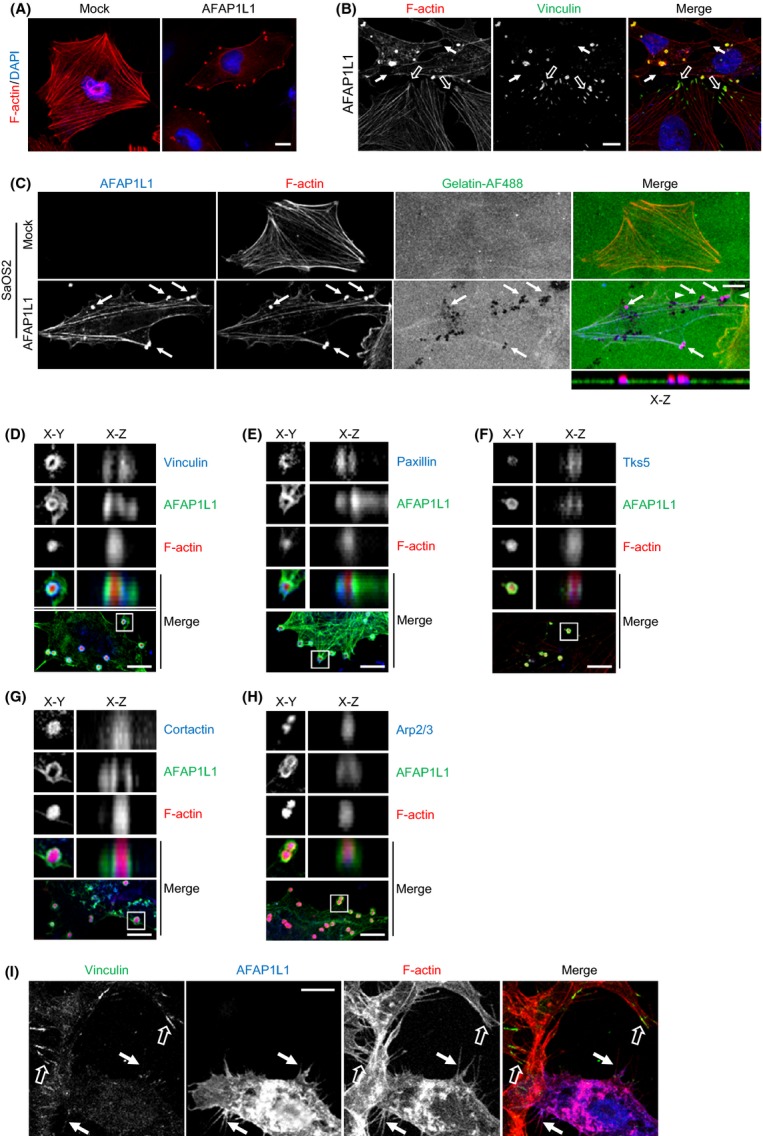
Localization of AFAP1L1 with component proteins in the invadopodia. (A–H) SaOS2 cells were transiently transfected with a mock or Flag-tagged AFAP1L1 expression plasmid, and were then stained at 24 h after transfection. (A, B) The cells were stained with rhodamine-conjugated phalloidin (F-actin, red), vinculin (green, except for [A]) and DAPI (blue). (A) Distribution of F-actin. In the AFAP1L1-transfected cells, the stress fibers were disassembled and the F-actin was aggregated into dots formed at the cell periphery. (B) Disassembly of focal adhesions. In the AFAP1L1-transfected cells with actin-rich dots, vinculin was detached from the focal adhesions at the tip of the actin stress fibers (solid arrows) as compared to the mature focal adhesions in the nontransfected cells (open arrows). (C) Colocalization of actin-rich dots with gelatin degradation spots. The cells were seeded onto AF488-conjugated gelatin (gelatin-AF488). The areas of gelatin degradation appear as fluorescence-negative spots beneath the cells. The arrows indicate newly formed invadopodia, which were merged with the gelatin degradation spots. The *x*–*z* plane image obtained between the two arrowheads shows the depth of the actin-rich protrusion invading into the gelatin substrate. (D–H) Localization and distribution of AFAP1L1 and other molecules in the invadopodia. The cells were stained for the indicated component proteins of the invadopodia (blue), Flag (AFAP1L1, green) and rhodamine-conjugated phalloidin (red). The upper four panels are magnified X–Y and X–Z plane images of the boxed areas in the lowest panel. (I) RKO cells were transiently transfected with a 3×Flag-tagged AFAP1L1 expression plasmid, and seeded onto FN-coated glass slides. The cells were then immunostained at 24 h after transfection for the indicated component proteins in the invadopodia (green), Flag (AFAP1L1, blue), and rhodamine-conjugated phalloidin (F-actin, red). The solid arrows indicate the detachment of vinculin from the focal adhesions as compared to the nontransfected cells (open arrows). The localization of each protein and the degradation of gelatin-AF488 are shown in the grayscale individually, as well as in each indicated color on the merged images, and counterstained for the nuclei with DAPI in (A) and (B). Scale bars: 10 *μ*m.

AFAP1L1 has been reported to be localized to the invadopodia, along with cortactin, in breast cancer cells 12. To further elucidate the molecular mechanism of AFAP1L1 in the regulation of cell shape and motility, we first reconfirmed the localization of AFAP1L1, together with several component proteins, in the invadopodia. SaOS2 cells transiently expressing AFAP1L1 had several podosome-like actin-rich dots, which were seldom observed in the mock-transfected cells (Fig.4A). In most of these cells, the focal adhesion structures were disassembled, and actin stress fibers were also disorganized (Fig.4B). When cultured on a fluorescence-conjugated gelatin substrate, these dots were involved in the degradation of the extracellular matrix (ECM) (Fig.4C), and thus we regarded these structures as invadopodia. Overexpressed AFAP1L1 was clustered in these invadopodia (Fig.4C), which is consistent with a previous report 12. Similarly, endogenous AFAP1L1 was detected exactly on the ECM-degrading invadopodia in U2OS cells (Fig. S6A). We further analyzed whether AFAP1L1 was colocalized with several other components of the invadopodia, including vinculin, paxillin, Tks5, cortactin, and actin-related protein 2/3 complex (Arp2/3) in SaOS2 cells (Fig.4D–H). AFAP1L1, vinculin, paxillin, and Tks5 displayed a ringed structure, whereas cortactin, Arp2/3, and F-actin seemed to form a solid core within it. The diameter of the ringed structure together with the solid core was approximately 2 *μ*m maximum, which was consistent with the average size of an invadopodium with a ringed structure estimated in previous reports 22,23. Thus, the whole structure was presumed to be a single invadopodium rather than a rosette consisting of fused invadopodia, and AFAP1L1 was localized to the ringed structure.

In AFAP1L1-transfected RKO cells, a dislocation of vinculin and paxillin from the focal adhesions, and the colocalization of AFAP1L1 with cortactin, Arp2/3, and F-actin, were observed (Figs.4I and S6B–D) as in AFAP1L1-transfected SaOS2 cells (Fig.4B). Therefore, RKO cells shared a common phenotype with SaOS2 cells with regard to the disassembly of focal adhesions when overexpressing AFAP1L1. We could not, however, detect either the formation of invadopodia nor ECM degradation. These results may be due to a lack of essential signal transduction or integral components required to form functional invadopodia in this cell line.

### AFAP1L1 was associated with vinculin

**Figure 5:**
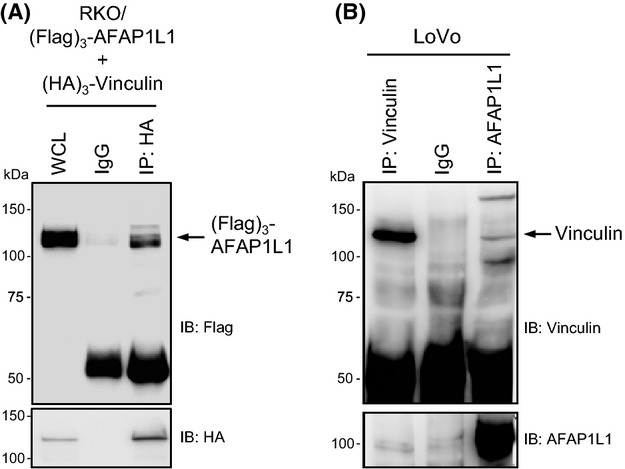
AFAP1L1 associates with vinculin. (A) Association of exogenous AFAP1L1 with vinculin. RKO cells were transiently transfected with both 3×Flag-tagged AFAP1L1 and 3×HA-tagged vinculin expression plasmids, and the cell lysates were immunoprecipitated with an anti-HA Ab and then immunoblotted with an anti-Flag Ab. A whole cell lysate was loaded as a positive control. (B) Association of endogenous AFAP1L1 with vinculin. LoVo-derived whole cell lysates were used to detect the interactions between the endogenous AFAP1L1 and vinculin proteins by immunoprecipitation with subsequent immunoblotting. The lysate immunoprecipitated with an anti-vinculin Ab was loaded as a positive control.

These results prompted us to examine the possibility that AFAP1L1 exerts a regulatory effect on cell shape and motility together with a novel interacting partner. We hypothesized that vinculin was the most likely candidate, as vinculin was colocalized with AFAP1L1 in the ringed structure of the invadopodia. In addition, our observations in the AFAP1L1-transduced cells as described above were similar to the alterations in cell shape and motility in vinculin-depleted cells 24,25. We hypothesized that AFAP1L1 would negatively modulate the integral function of vinculin. To examine whether AFAP1L1 would form a complex with vinculin, coimmunoprecipitation experiments were carried out. First, in RKO cells expressing both flag-tagged AFAP1L1 (Flag-AFAP1L1) and HA-tagged vinculin (HA-vinculin), Flag-AFAP1L1 was found to be coimmunoprecipitated with HA-vinculin using an anti-HA Ab (Fig.5A). In addition, the direct interaction between endogenous AFAP1L1 and vinculin was confirmed in LoVo cells using anti-AFAP1L1 and anti-vinculin Abs (Fig.5B). We did not detect the coimmunoprecipitation of endogenous AFAP1L1 with endogenous paxillin, cortactin, talin, tks5, FAK, p130Cas, or Src (Fig. S7), although overexpressed AFAP1L1 was reported to associate with overexpressed cortactin 12. These results strongly support the interaction of AFAP1L1 with vinculin.

### AFAP1L1-transduced cells were resistant to anoikis

**Figure 6:**
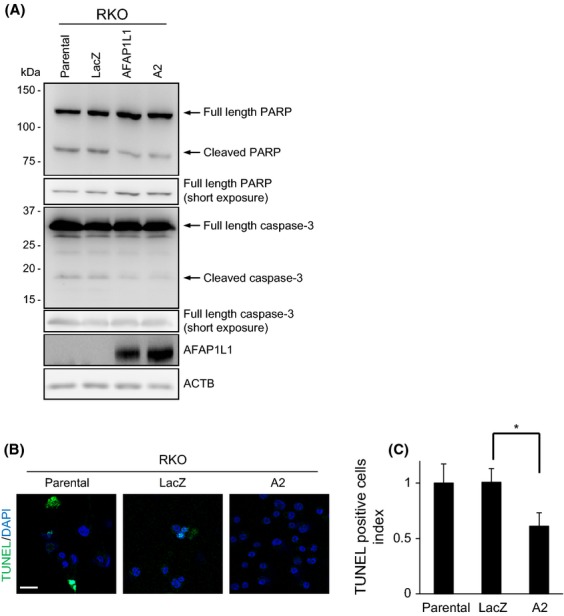
AFAP1L1-transduced RKO cells are resistant to anoikis. Stable RKO-derived cells were cultured for 72 h in suspension. (A) Whole cell lysates were immunoblotted with an anti-cleaved PARP Ab or an anti-caspase 3 Ab. The shorter exposure images of the full-length PARP and caspase 3 show the equality of the protein loading volume. (B, C) The cells were then smeared on glass slides and labeled by TUNEL (green). The nuclei were counterstained with DAPI (blue) to count the cells. Representative images are shown in (B), and the mean proportion of TUNEL-positive cells in 10 random view fields are presented in (C) as an index relative to the parental RKO cells. *P < 0.05 by two-sided Student's *t*-test. Scale bar: 20 *μ*m.

Vinculin-null F9 embryonic mouse carcinoma cells are known to be resistant to apoptosis induced by detachment from the cellular substrate (anoikis) or by serum withdrawal 26. To test our hypothesis that AFAP1L1 would be a functional regulator of vinculin, we finally examined whether AFAP1L1 could alter the apoptotic status. When RKO-derived cells were cultured for 72 h in suspension or in serum-depleted media, the cleavage of both caspase-3 and poly-ADP ribose polymerase (PARP) were clearly reduced in RKO/AFAP1L1 and A2 cells cultured in suspension, and a slight inhibition of PARP cleavage was also observed upon serum deprivation (Figs.6A, S8). Anoikis resistance was further confirmed by a decrease in the TUNEL-positive rates in A2 cells (Fig.6B and C). This result suggests the possibility that AFAP1L1 could inhibit anoikis through interactions with vinculin.

## Discussion

AFAP1L1 is a member of the AFAP family of three proteins, which also includes AFAP1 and AFAP1L2/XB130. Among these proteins with structurally homologous protein-binding motifs, AFAP1, also known as AFAP-110, is a well-studied Src-binding partner 13,21,27. It has the capability to alter actin filament integrity, and is involved in the dynamics of the invadopodia 28–30. AFAP1 and AFAP1L2/XB130 associate with Src via their SH3-binding motifs 21,31. In contrast, recent evidence suggests that AFAP1L1 does not bind strongly to Src, but rather associates with cortactin, and that AFAP1L1 and cortactin colocalize within the invadopodia 12.

In this study, we demonstrated the possible interaction of AFAP1L1 with vinculin. Vinculin is an adaptor protein which localizes to integrin-mediated cell-matrix adhesions such as focal adhesions and invadopodia 32,33. It stabilizes these focal adhesions and has been described as a suppresser of cellular migration 34,35. The loss of vinculin expression is likely to endow these cells with metastatic potential 33. Indeed, low levels of vinculin expression are related to metastatic spread in squamous cell carcinomas 36. Furthermore, the knocking-out of vinculin expression altered the phenotype drastically in F9 and mouse embryonic fibroblast cells. These vinculin-null cells showed a rounded shape, and had smaller focal adhesion complexes with increased motility on a planar substrate 24,25, which bear close resemblance to the AFAP1L1-transduced RKO cells in this study. We showed the colocalization of overexpressed AFAP1L1 with vinculin in invadopodia, and also the possible interaction of endogenous AFAP1L1 with vinculin. Collectively, these results strongly support the hypothesis that the AFAP1L1-induced alterations in cell shape and motility are mediated through its interactions with vinculin. In contrast, it is well-established that Rho-GTPases definitely play a critical role in the regulation of the actin cytoskeleton, including the cell shape and migration 6,37–40. However, Rho-GTPases are regulated by numerous proteins, and their precise regulatory mechanisms are not yet fully understood. Several reports have demonstrated that vinculin function is also influenced by Rho-GTPases 41,42. Further studies are needed to elucidate the detailed regulatory pathway involving AFAP1L1, vinculin and Rho-GTPases.

Invadopodia are highly dynamic protrusions at the substrate-contacting side of the cell 43, and are involved in the degradation of the ECM 44. We have reported that the ectopic expression of AFAP1L1 in immortalized mesenchymal stem cells resulted in enhanced migration in matrix gels 11.

In the present study, the ectopic overexpression of AFAP1L1 induced the detachment of vinculin from the focal adhesions in both RKO and SaOS2 cells, which probably reflected the disassembly of these structures. The disassembly of focal adhesions can lead to alterations in both cell shape and motility, presumably as a consequence of reduced cell-matrix contacts 35, and it also generally coincides with the dynamic formation of podosomes/invadopodia 22,45. We previously showed a significant decrease in matrix invasion after AFAP1L1 knocking-down in U2OS cells 11. In this study, we failed to observe clear invadopodia in the CRC cells employed. In addition, there were no significant effects of AFAP1L1 on the invasion capability of CRC cells using a conventional assessment with Boyden-chambers (data not shown). Thus, AFAP1L1 does not seem to contribute to the matrix-degrading invasion in the CRC cells used in this study. We have no clear explanation for the difference between sarcoma cells and CRC cells. We speculate that the basic physiologic function of AFAP1L1 may be to modulate cell-matrix interactions through vinculin, and that this effect may result in different cellular phenotypes in different types of cells, such as sarcoma and carcinoma cells, which have different intrinsic characteristics in terms of their cell-matrix interactions 46. The discrepancy between the insufficient knocking-down effect on cell motility and the significant attenuation of in vivo growth in LoVo cells remains unknown. AFAP1L1 might play a more critical role in cell survival in the in vivo environment as compared to in vitro culture.

It is intriguing that the AFAP1L1-transduced cells became resistant to anoikis. There is an open question from the previous study and from this study about the striking difference in the effects of AFAP1L1 on cell growth in vitro versus its matched xenograft in vivo. We speculate that this may reflect the extent of anoikis induced by some extracellular stimuli. Subcutaneous injections of a cell suspension in PBS are highly likely to evoke a significant increase in anoikis-related signaling cascades. Nevertheless, due to the acquisition of resistance against anoikis, AFAP1L1-expressing cells might survive this critical step, especially at the very beginning of xenograft formation, and consequently grew faster. Collectively, we hypothesized that the adverse impact of upregulated AFAP1L1 expression on the treatment outcomes of CRCs might be attributable to enhanced cell motility and increased anoikis resistance, rather than to enhanced invasion capability through the degradation of the surrounding matrix.

In conclusion, *AFAP1L1* gene expression was upregulated in CRC cells, and targeted treatment against AFAP1L1 was found to be effective in a mouse xenograft model. In the multivariate logistic regression analysis for recurrence in patients with rectal cancers, strong AFAP1L1 protein expression was found to be an independent and significant factor. AFAP1L1 can modulate cell shape and motility, presumably partially through interactions with vinculin, and may play a role in the inhibition of anoikis. However, the mechanism by which AFAP1L1 regulates the function of vinculin remains unknown. With regard to the regulation of *AFAP1L1* gene expression, we demonstrated that the binding motifs of one of the major transcriptional factors, specificity protein 3 (Sp3), which is located upstream of the *AFAP1L1* gene transcription start site, are essential for the induction of *AFAP1L1* gene expression 47. There are several new and intriguing reports that propose a role for AFAP1L1 in the initiation and progression of CRCs. Using genome-wide mRNA expression profiling and mass spectrometry, the *AFAP1L1* gene was identified as one of the candidate genes specifically upregulated in a leucine-rich repeat containing G protein-coupled receptor 5 (Lgr5)-positive human intestinal stem cells (ISC) 48. In addition, the expression patterns of the ISC-specific genes, including the *AFAP1L1* gene, could predict disease relapses in CRC patients 49. Further investigation is underway to establish the importance of this protein as a molecular biomarker, and as a therapeutic target for CRCs and to uncover its role in cancer progression.

## Supporting Information

**Figure S1**. Differential expression patterns of AFAP family members in CRC tissues. (A) The expression levels of the *AFAP1L1* gene were assessed by qRT-PCR using the first set of 33 CRC tissues. The ratios of *β-actin-*normalized expression in the tumors relative to that of the matched normal mucosa were presented in base two logarithm (log_2_[T/N]). (B) The correlation between the *β-actin-*normalized T/N ratio (*x*-axis) with the *β-2-microglubulin*-normalized ratio (*B2M*, *y*-axis) in all 61 samples is shown with the fitted linear regression. (C–F) Using the same 33 sample pairs as in (A), the *AFAP1* (C) and *AFAP1L2* (E) gene expression levels were also analyzed by qRT-PCR. The correlation between the expression level of the *AFAP1* (D) or *AFAP1L2* (F) gene with the *AFAP1L1* gene in these 33 tumor samples is shown with the fitted linear regression. In (A, C, and E), the ratios of the *β-actin-*normalized expression in the tumors relative to the matched normal mucosa were presented in base two logarithm (log_2_[T/N]). The dashed line indicates log_2_[T/N] = 1. The *R* value of Pearson's correlation coefficient was also calculated and presented in (B), (D), and (F).

**Figure S2.** In vitro and in vivo cell growth. (A) 13 CRC cell lines were analyzed for AFAP1L1 expression by RT-PCR and western immunoblotting. (B) The expression levels of AFAP1L1 in SW480 cells stably expressing AFAP1L1 were determined by RT-PCR and immunoblotting. LacZ-transduced cells were used as a control. (C) Growth curves of subcutaneous xenografts derived from SW480 cells stably expressing AFAP1L1 in nude mice (*n* = 4 per group). (D) In vitro growth curves of stable LoVo-derived cells used in Figure D. The indicated cell lines were cultured on 60 mm dishes and were counted with a hemocytometer every 24 h. The data are presented as means ± SE. **P* < 0.05 by two-sided Student's *t*-test.

**Figure S3.** Quantification of the signal intensity in immunoblotting. The signal intensities of the AFAP1L1 protein after immunoblotting in Figure D (A), F (B), and I (C) were quantified and normalized against *β*-actin. The results are presented as a ratio relative to the respective control sample.

**Figure S4.** The effects of AFAP1L1 expression on cellular morphology and motility are not fibronectin-dependent. The proportion of rounded cells (A) and the mean speed of cellular movement (B) of the indicated stable RKO-derived cell lines on either FN-coated or noncoated dishes were measured. The same data as in Figure B and C in the FN-coated group are also shown for comparison. (C) The persistence of cellular movement in each cell line and their conditions were calculated by dividing the total distance migrated by the net distance migrated. The *P* value was calculated by a two-sided Student's *t*-test. The data are presented as means ± SE. **P* < 0.05; ***P* < 0.01 by two-sided Student's *t*-test; N.S., not significant.

**Figure S5.** The effects of AFAP1L1 expression on cellular morphology and motility are not correlated with Src activation status. (A) Cell lysates from stable RKO cells were analyzed for phosphorylation at Src tyrosine 416 (Src pY416) by immunoblotting. The expression level of total Src is shown as a loading control. (B) RKO cells were transiently transfected with mock, AFAP1L1 or AFAP1 expression plasmids, and then the cell lysates were analyzed for Src pY416. (C–E) Stable RKO-derived cells were treated with 10 *μ*mol/L PP2 for 4 h. (C) The attenuation of Src pY416 after the PP2 treatment was confirmed by immunoblotting. The changes in cellular morphology were quantified in (D), and representative phase contrast images are shown in (E). The data are presented as means ± SE. **P* < 0.05 by two-sided Student's *t*-test.

**Figure S6.** Localization of AFAP1L1 with component proteins in the invadopodia. (A) Localization of endogenous AFAP1L1 in the invadopodia. U2OS cells were seeded onto gelatin-AF488 for 24 h, and then immunostained with an anti-AFAP1L1 Ab (blue) and rhodamine-conjugated phalloidin (red). An arrow indicates the complete merge of the aggregated invadopodia with the gelatin degradation spots, and also with endogenous AFAP1L1 expression. The lower panels are magnified views of the boxed area in the upper panels. *, nonspecific immunostaining of the nuclei by an anti-AFAP1L1 Ab. (B–D) RKO cells were transiently transfected with a 3×Flag-tagged AFAP1L1 expression plasmid and seeded onto FN-coated glass slides. The cells were then immunostained at 24 h after transfection with the indicated component proteins for the invadopodia (green), Flag (AFAP1L1, blue), and rhodamine-conjugated phalloidin (F-actin, red). The solid arrows in (B) indicate the detachment of paxillin from the focal adhesions as compared to the nontransfected cells (open arrows). The arrows in (C) and (D) indicate the aggregation of cortactin (C) or Arp2/3 (D) on the ventral side of the cell surface, which are merged with the signals from both AFAP1L1 and F-actin. The localization of each protein is shown in the grayscale individually, as well as in each indicated color on the merged images. Scale bars: 10 *μ*m.

**Figure S7.** AFAP1L1 is not coprecipitated with other component proteins in the invadopodia or the focal adhesions. LoVo-derived whole cell lysates were immunoprecipitated with the anti-AFAP1L1 Ab as in Figure D, and subsequently immunoblotted with the indicated Abs against component proteins in the invadopodia or focal adhesions. A whole cell lysate (WCL) was loaded as a positive control. *: nonspecific band.

**Figure S8.** AFAP1L1-transduced RKO cells are resistant to apoptosis induced by serum deprivation. Stable RKO-derived cells were cultured for 72 h in serum-depleted media. The whole cell lysates were immunoblotted with an anti-cleaved PARP Ab.

**Table S1.** Antibodies and Taqman probes used in the study. The anti-AFAP1L1 polyclonal Ab was produced in our laboratory. The other Abs were purchased from the indicated companies. Taqman probes were purchased from Applied Biosystems (Carlsbad, CA). IHC, immunohistochemistry; IF, immunofluorescence; WB, western blotting; IP, immunoprecipitation.

**Table S2.** Mean threshold cycle (Ct) values of frozen samples from CRC patients. The mean Ct values for the *AFAP1L1*, *β-actin,* and *B2M* gene in the tumors and the adjacent normal mucosa from the 61 CRC patients analyzed by qRT-PCR in this study are listed.

**Table S3.** Demographics of the CRC patients for immunohistochemical analysis. The patient profiles of the 164 CRC specimens evaluated by immunohistochemistry in this study. SD, standard deviation.

**Table S4.** Correlation between clinicopathologic features and AFAP1L1 expression levels. The expression intensity was compared among various clinicopathologic factors. *χ*^2^ test, chi-square test.

**Table S5.** Logistic regression analysis for recurrence in curative resections for rectal cancers. Multivariate logistic regression analysis for recurrence in the 80 rectal cancers. AFAP1L1, actin filament-associated protein 1-like 1; pN, pathological nodal stage; M, male; F, female.

**Movie S1.** Motility of stable RKO-derived cells on a 2D substrate. Stable RKO-derived cells were seeded onto FN-coated dishes, and their movements were captured by time-lapse microscopy every 4 min for 6 h (parental RKO cells).

**Movie S2.** Motility of stable RKO-derived cells on a 2D substrate. Stable RKO-derived cells were seeded onto FN-coated dishes, and their movements were captured by time-lapse microscopy every 4 min for 6 h (RKO/LacZ cells).

**Movie S3.** Motility of stable RKO-derived cells on a 2D substrate. Stable RKO-derived cells were seeded onto FN-coated dishes, and their movements were captured by time-lapse microscopy every 4 min for 6 h (RKO/AFAP1L1 cells).

**Movie S4.** Motility of stable RKO-derived cells on a 2D substrate. Stable RKO-derived cells were seeded onto FN-coated dishes, and their movements were captured by time-lapse microscopy every 4 min for 6 h (A2 cells).
