# Supplementary material for: AFAP1L1, a novel associating partner with vinculin, modulates cellular morphology and motility, and promotes the progression of colorectal cancers
Source: Cancer Med. 2014 Apr 10;3(4):759–74. doi: 10.1002/cam4.237 (PMC4303145; doi:10.1002/cam4.237)
Supplement: Supplementary file 1 [file cam40003-0759-sd1.ppt]

## Slide 1
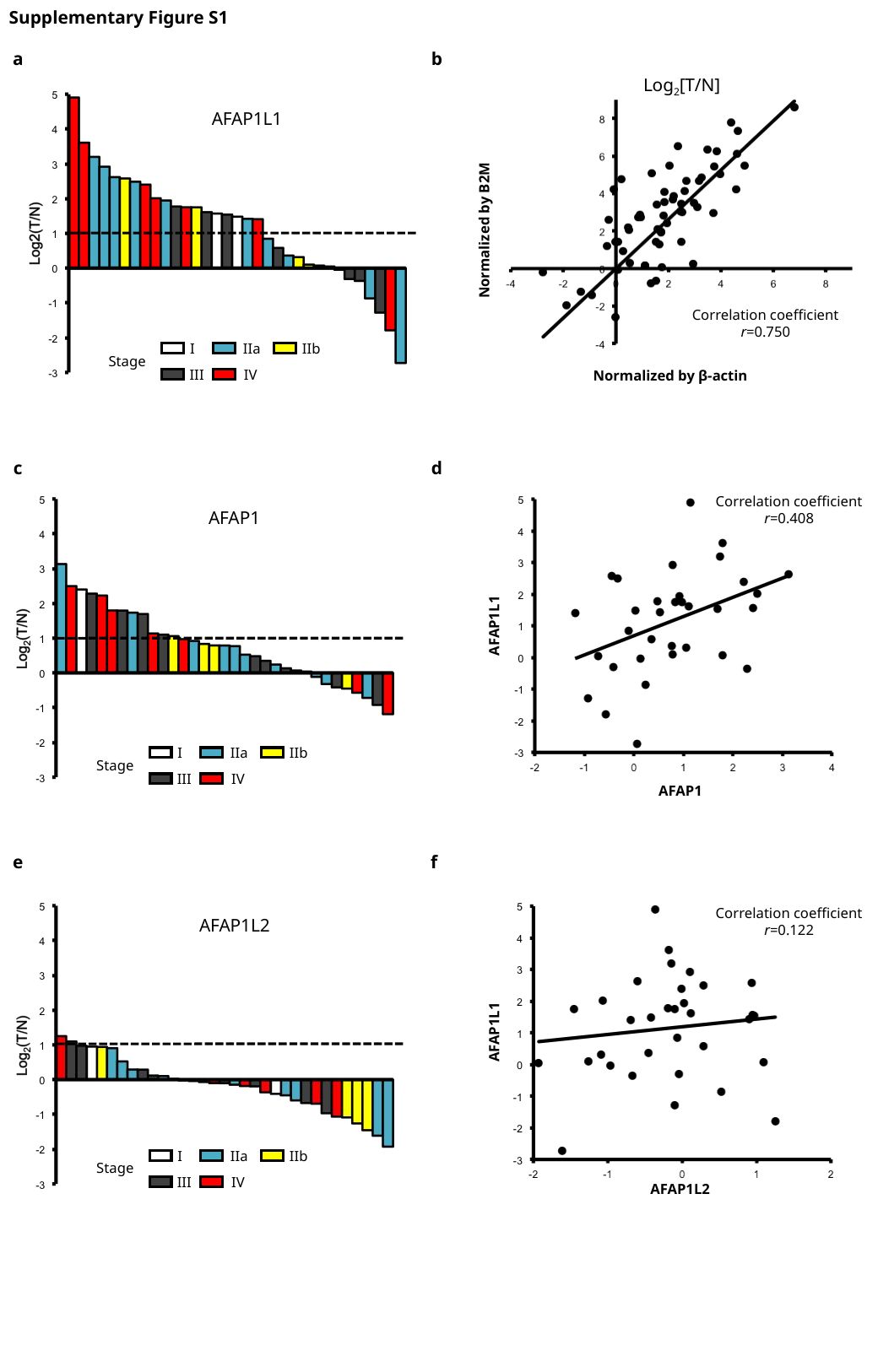

Supplementary Figure S1
a
b
Log2[T/N]
Normalized by B2M
Correlation coefficient
r=0.750
Normalized by β-actin
AFAP1L1
I
IIa
IIb
Stage
III
IV
c
d
AFAP1
I
IIa
IIb
Stage
III
IV
AFAP1L1
AFAP1
Correlation coefficient
r=0.408
e
f
AFAP1L2
I
IIa
IIb
Stage
III
IV
AFAP1L1
AFAP1L2
Correlation coefficient
r=0.122
