# Supplementary material for: AFAP1L1, a novel associating partner with vinculin, modulates cellular morphology and motility, and promotes the progression of colorectal cancers
Source: Cancer Med. 2014 Apr 10;3(4):759–74. doi: 10.1002/cam4.237 (PMC4303145; doi:10.1002/cam4.237)
Supplement: Supplementary file 3 [file cam40003-0759-sd3.ppt]

## Slide 1
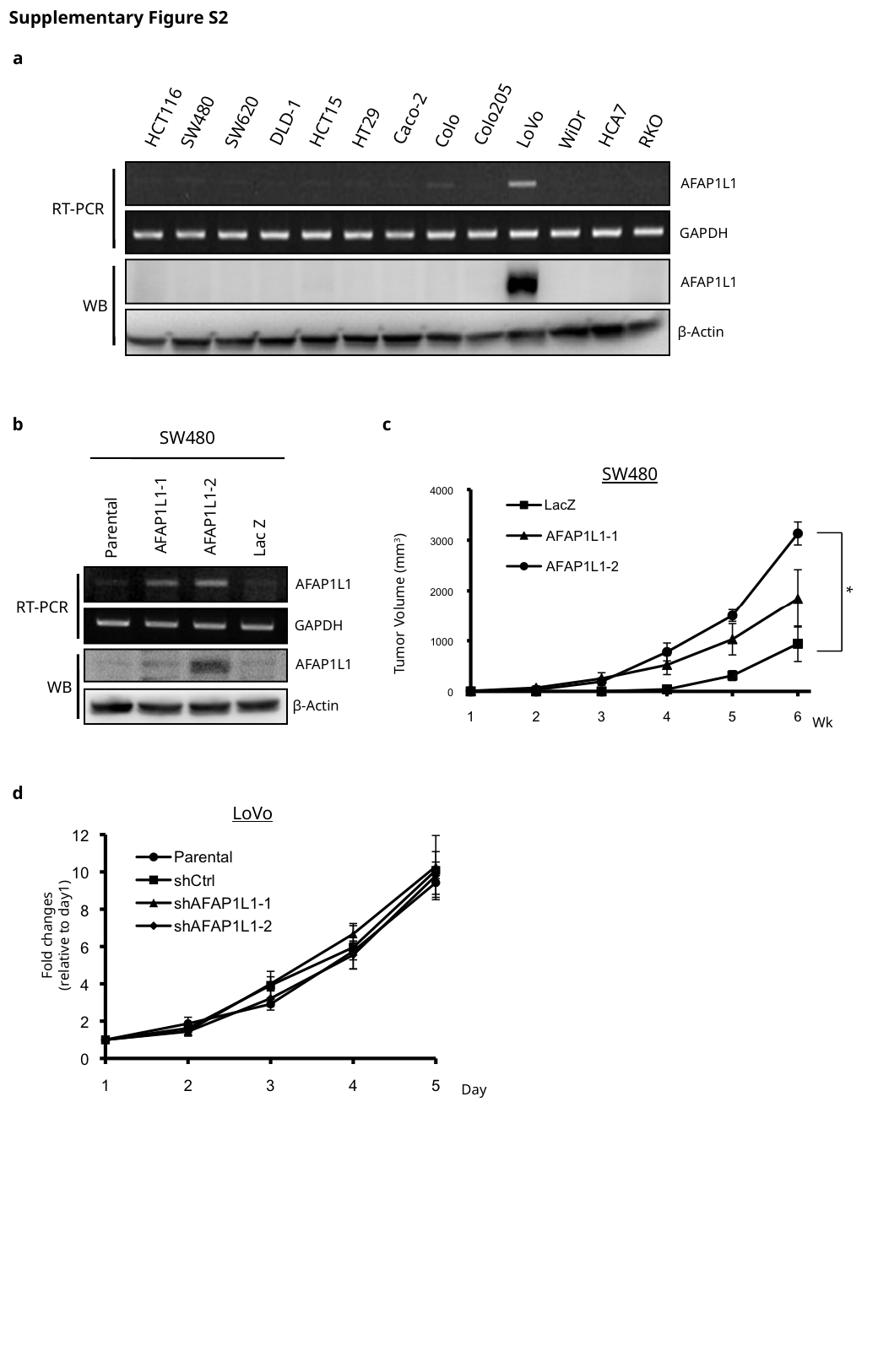

Supplementary Figure S2
a
Colo205
HCT116
Caco-2
HCT15
SW620
DLD-1
SW480
HCA7
HT29
LoVo
WiDr
RKO
Colo
AFAP1L1
GAPDH
AFAP1L1
β-Actin
RT-PCR
WB
b
c
SW480
AFAP1L1-1
AFAP1L1-2
Parental
Lac Z
AFAP1L1
GAPDH
AFAP1L1
β-Actin
RT-PCR
WB
SW480
*
Tumor Volume (mm3)
Wk
d
LoVo
Fold changes
(relative to day1)
Day
