# Supplementary figures and images for: AFAP1L1, a novel associating partner with vinculin, modulates cellular morphology and motility, and promotes the progression of colorectal cancers
Source: Cancer Med. 2014 Apr 10;3(4):759–74. doi: 10.1002/cam4.237 (PMC4303145; doi:10.1002/cam4.237)

## Slide 1
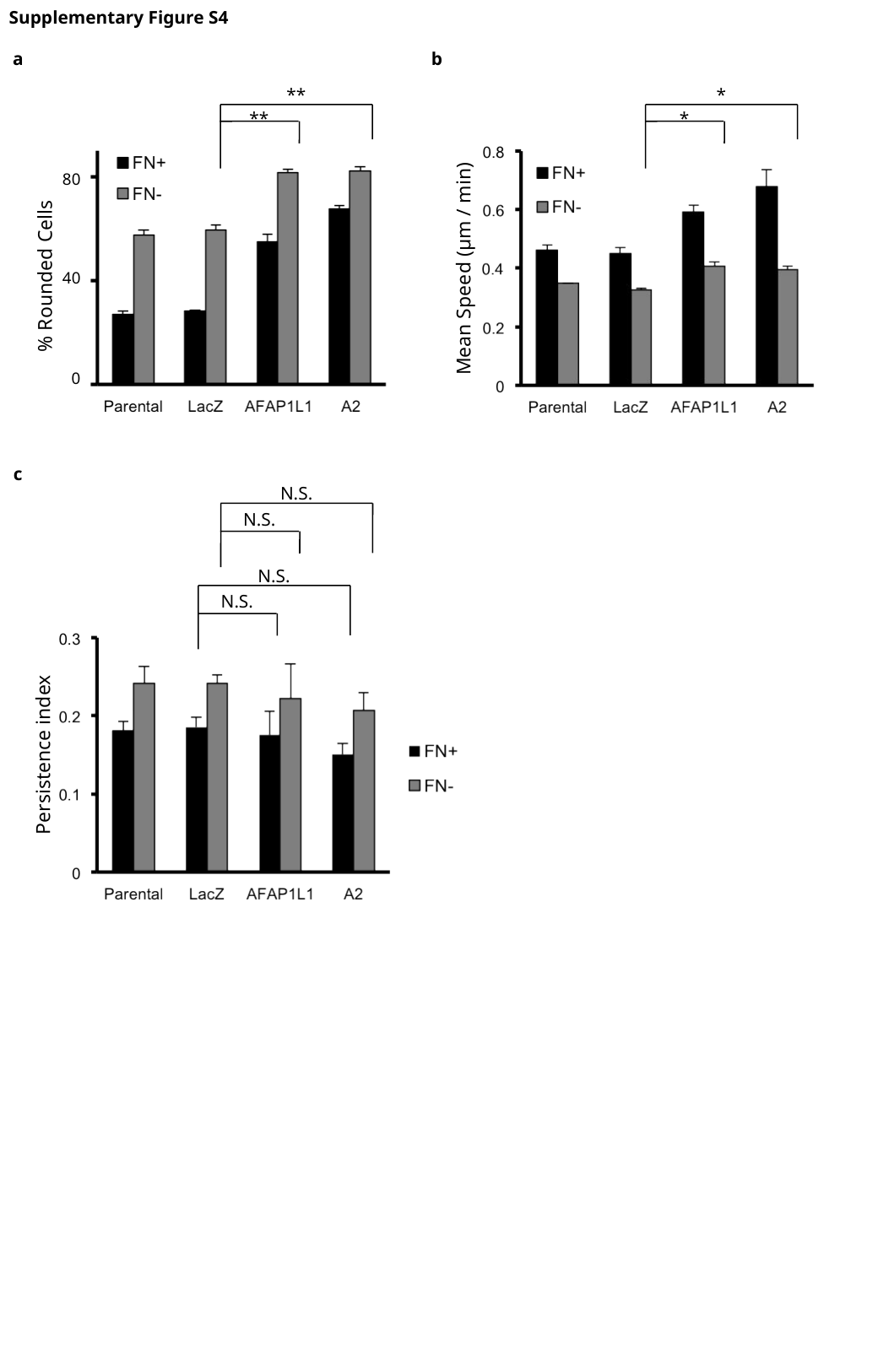

Supplementary Figure S4
a
b
**
**
80
% Rounded Cells
40
0
*
*
Mean Speed (µm / min)
c
N.S.
N.S.
N.S.
N.S.
Persistence index

Supplement: Supplementary file 4 [file cam40003-0759-sd4.ppt]

## Slide 1
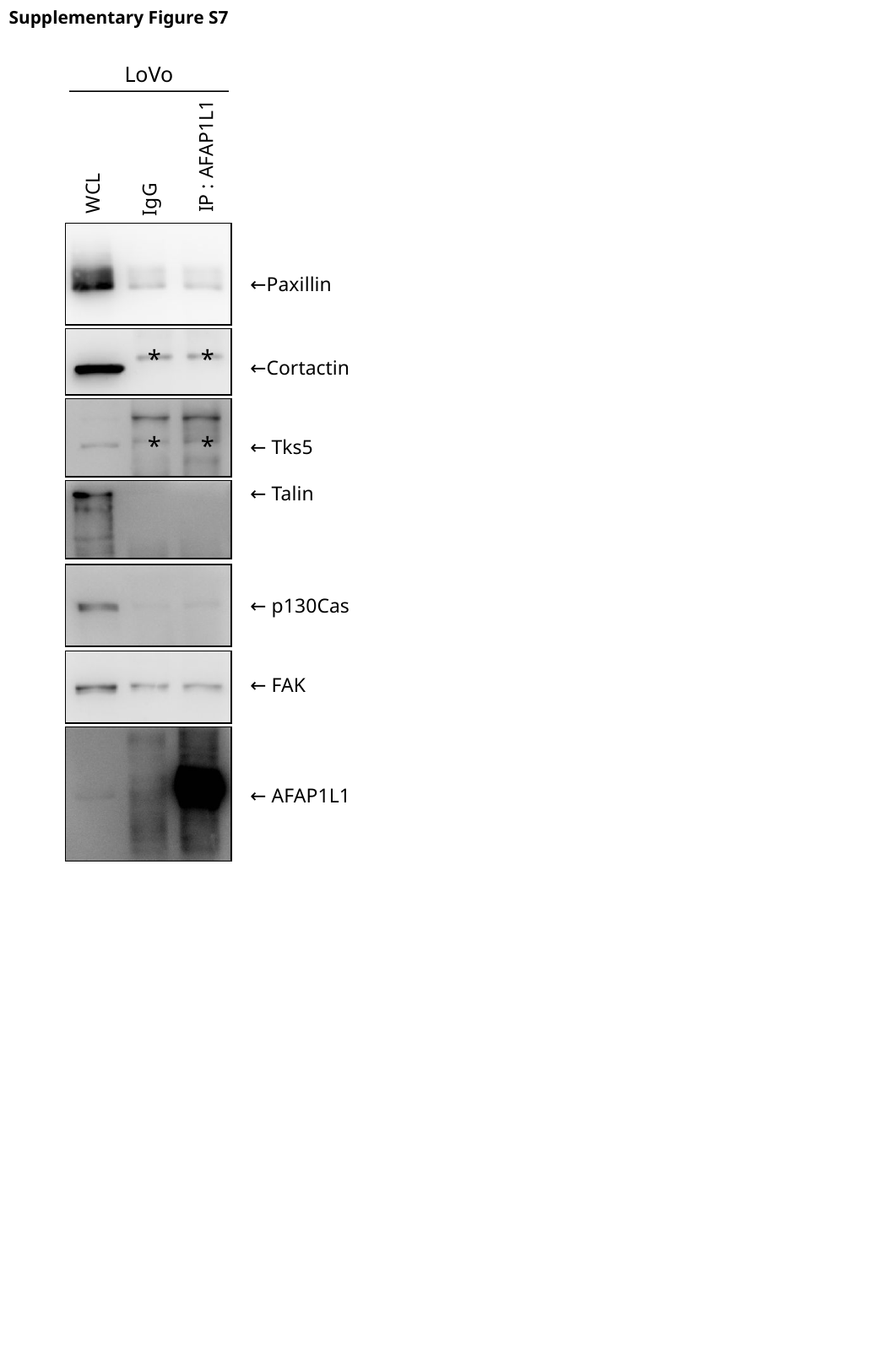

Supplementary Figure S7
LoVo
IP : AFAP1L1
WCL
IgG
←Paxillin
*
*
←Cortactin
*
*
← Tks5
← Talin
← p130Cas
← FAK
← AFAP1L1

Supplement: Supplementary file 7 [file cam40003-0759-sd7.ppt]
