# Supplementary material for: AFAP1L1, a novel associating partner with vinculin, modulates cellular morphology and motility, and promotes the progression of colorectal cancers
Source: Cancer Med. 2014 Apr 10;3(4):759–74. doi: 10.1002/cam4.237 (PMC4303145; doi:10.1002/cam4.237)
Supplement: Supplementary file 5 [file cam40003-0759-sd5.ppt]

## Slide 1
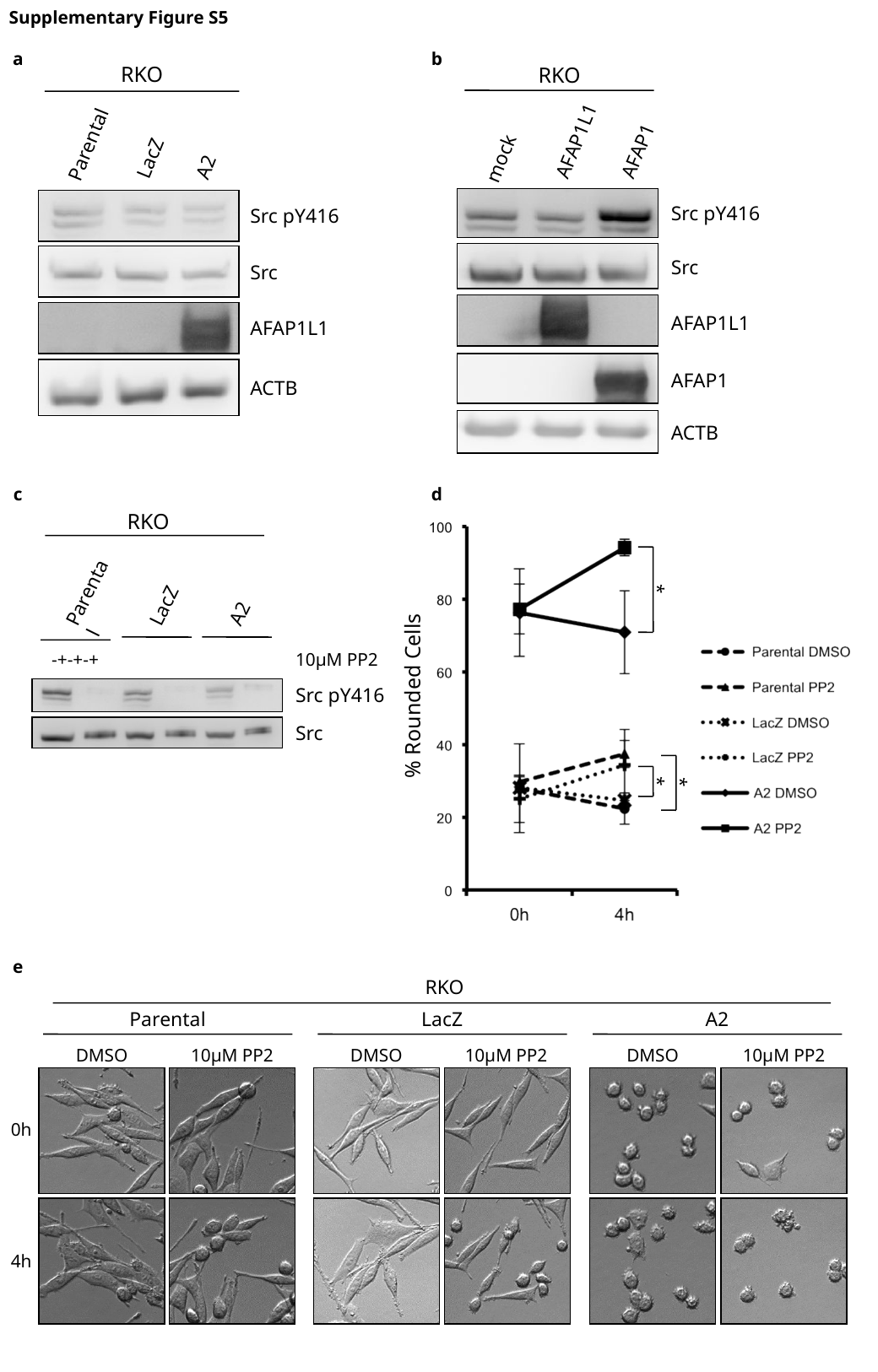

Supplementary Figure S5
a
b
RKO
Parental
LacZ
A2
Src pY416
Src
AFAP1L1
ACTB
RKO
AFAP1L1
AFAP1
mock
Src pY416
Src
AFAP1L1
AFAP1
ACTB
c
d
RKO
Parental
A2
LacZ
-+-+-+
10µM PP2
Src pY416
Src
% Rounded Cells
*
*
*
e
RKO
Parental
LacZ
A2
DMSO
10µM PP2
DMSO
10µM PP2
DMSO
10µM PP2
0h
4h
