# Supplementary material for: AFAP1L1, a novel associating partner with vinculin, modulates cellular morphology and motility, and promotes the progression of colorectal cancers
Source: Cancer Med. 2014 Apr 10;3(4):759–74. doi: 10.1002/cam4.237 (PMC4303145; doi:10.1002/cam4.237)
Supplement: Supplementary file 6 [file cam40003-0759-sd6.ppt]

## Slide 1
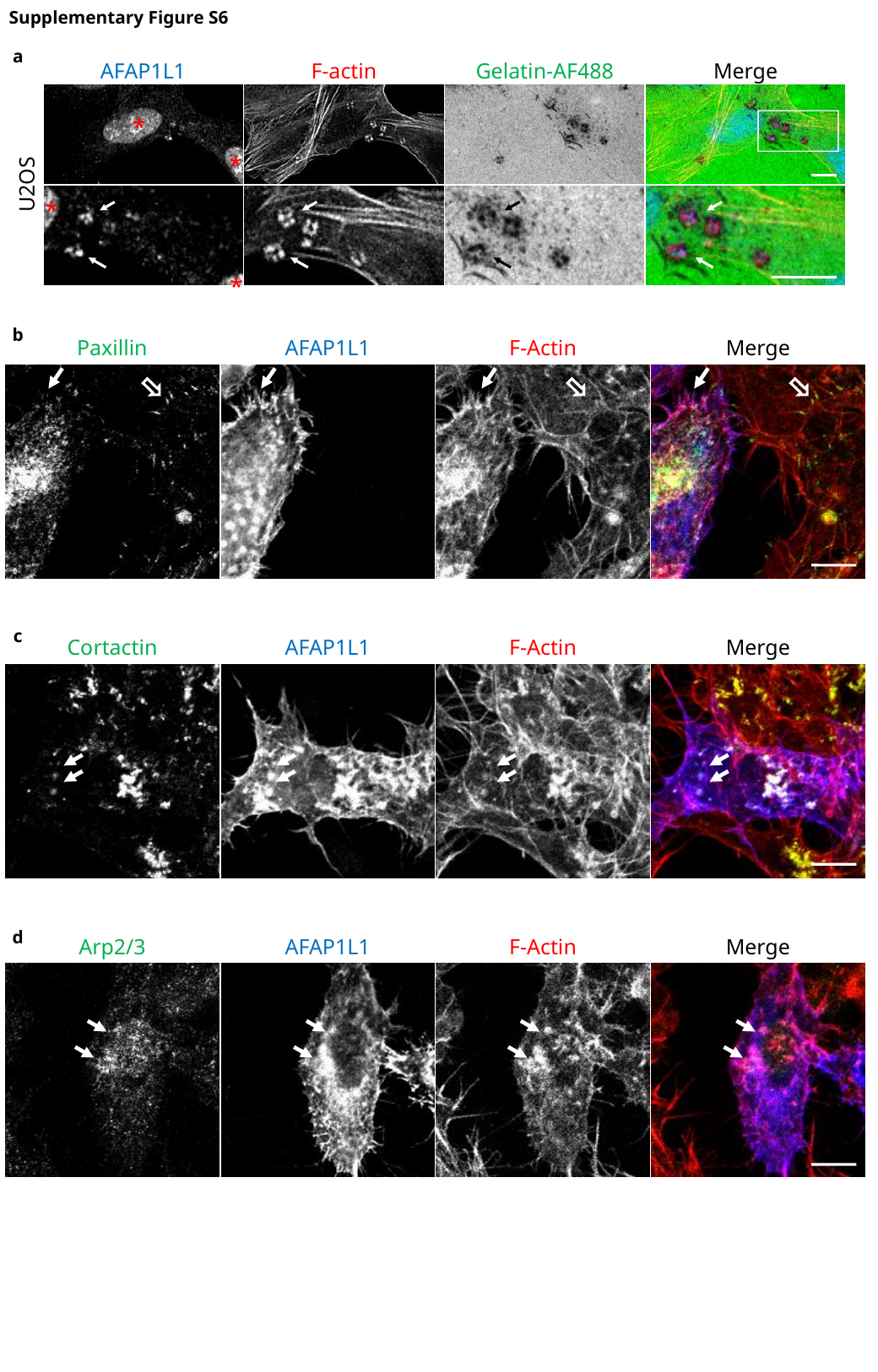

Supplementary Figure S6
a
AFAP1L1
F-actin
Gelatin-AF488
Merge
*
*
U2OS
*
*
b
Paxillin
AFAP1L1
F-Actin
Merge
c
Cortactin
AFAP1L1
F-Actin
Merge
d
Arp2/3
AFAP1L1
F-Actin
Merge
