# Supplementary material for: AFAP1L1, a novel associating partner with vinculin, modulates cellular morphology and motility, and promotes the progression of colorectal cancers
Source: Cancer Med. 2014 Apr 10;3(4):759–74. doi: 10.1002/cam4.237 (PMC4303145; doi:10.1002/cam4.237)
Supplement: Supplementary file 8 [file cam40003-0759-sd8.ppt]

## Slide 1
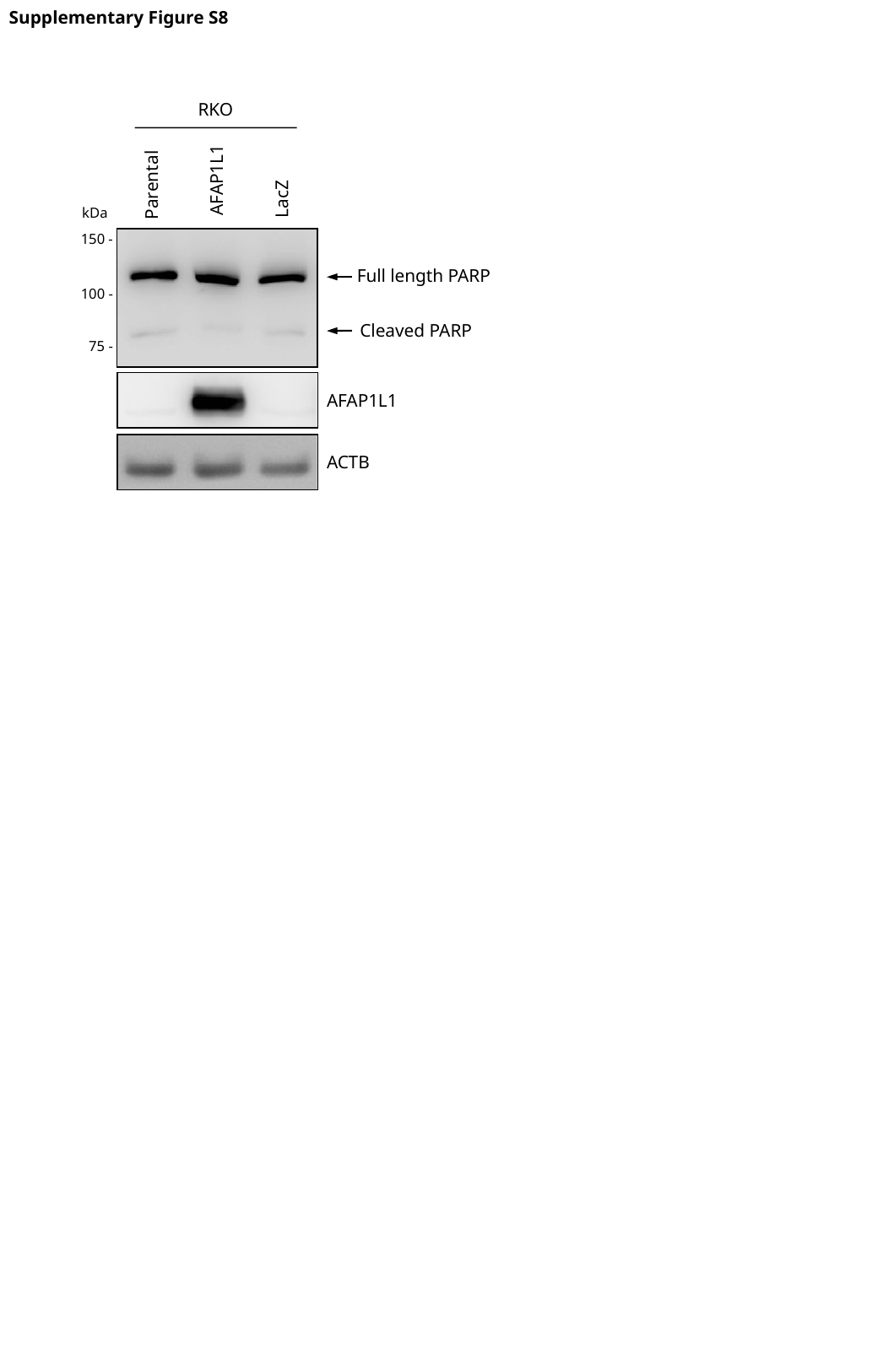

Supplementary Figure S8
RKO
AFAP1L1
Parental
LacZ
kDa
150 -
Full length PARP
100 -
Cleaved PARP
75 -
AFAP1L1
ACTB
